# Detecting and Characterizing Inferior Vena Cava Filters on Abdominal Computed Tomography with Data-Driven Computational Frameworks

**DOI:** 10.1007/s10278-023-00882-1

**Published:** 2023-09-28

**Authors:** Sema Candemir, Robert Moranville, Kelvin A. Wong, Warren Campbell, Matthew T. Bigelow, Luciano M. Prevedello, Mina S. Makary

**Affiliations:** 1 Department of Radiology, The Ohio State University Wexner Medical Center, Columbus, OH 43210 USA; 2 Laboratory for Augmented Intelligence in Imaging, The Ohio State University, Columbus, OH 43210 USA

**Keywords:** Data-driven computational frameworks, Machine learning, Radiology, Computed tomography, Inferior vena cava filters

## Abstract

Two data-driven algorithms were developed for detecting and characterizing Inferior Vena Cava (IVC) filters on abdominal computed tomography to assist healthcare providers with the appropriate management of these devices to decrease complications: one based on 2-dimensional data and transfer learning (2D + TL) and an augmented version of the same algorithm which accounts for the 3-dimensional information leveraging recurrent convolutional neural networks (3D + RCNN). The study contains 2048 abdominal computed tomography studies obtained from 439 patients who underwent IVC filter placement during the 10-year period from January 1st, 2009, to January 1st, 2019. Among these, 399 patients had retrievable filters, and 40 had non-retrievable filter types. The reference annotations for the filter location were obtained through a custom-developed interface. The ground truth annotations for the filter types were determined based on the electronic medical record and physician review of imaging. The initial stage of the framework returns a list of locations containing metallic objects based on the density of the structure. The second stage processes the candidate locations and determines which one contains an IVC filter. The final stage of the pipeline classifies the filter types as retrievable vs. non-retrievable. The computational models are trained using Tensorflow Keras API on an Nvidia Quadro GV100 system. We utilized a fine-tuning supervised training strategy to conduct our experiments. We find that the system achieves high sensitivity on detecting the filter locations with a high confidence value. The 2D + TL model achieved a sensitivity of 0.911 and a precision of 0.804, and the 3D + RCNN model achieved a sensitivity of 0.923 and a precision of 0.853 for filter detection. The system confidence for the IVC location predictions is high: 0.993 for 2D + TL and 0.996 for 3D + RCNN. The filter type prediction component of the system achieved 0.945 sensitivity, 0.882 specificity, and 0.97 AUC score with 2D + TL and 0. 940 sensitivity, 0.927 specificity, and 0.975 AUC score with 3D + RCNN. With the intent to create tools to improve patient outcomes, this study describes the initial phase of a computational framework to support healthcare providers in detecting patients with retained IVC filters, so an individualized decision can be made to remove these devices when appropriate, to decrease complications. To our knowledge, this is the first study that curates abdominal computed tomography (CT) scans and presents an algorithm for automated detection and characterization of IVC filters.

## Introduction

The field of diagnostic decision support in radiology has been rapidly progressing with the increasing availability of patient data, the development of machine learning (ML) techniques that extract detailed information from data, and powerful computers to efficiently run these ML techniques [1–3]. Multiple systems have been developed so far to assist radiologists, such as disease detection [4–8], identification of disease progression [9], and lesion localization [4, 10, 11]. This study investigates the performance of a three-stage data-driven computational framework to automatically detect inferior vena cava (IVC) filters on abdominal computed tomography (CT) and determine their subtype based on their morphology. This automated system may support radiologists in locating the filter, detecting the filter type, and flag incidentally found retrievable IVC filters on CT studies to populate a registry that the care team can then use to determine if and when the filters need to be removed to minimize complications.

**Fig. 1:**
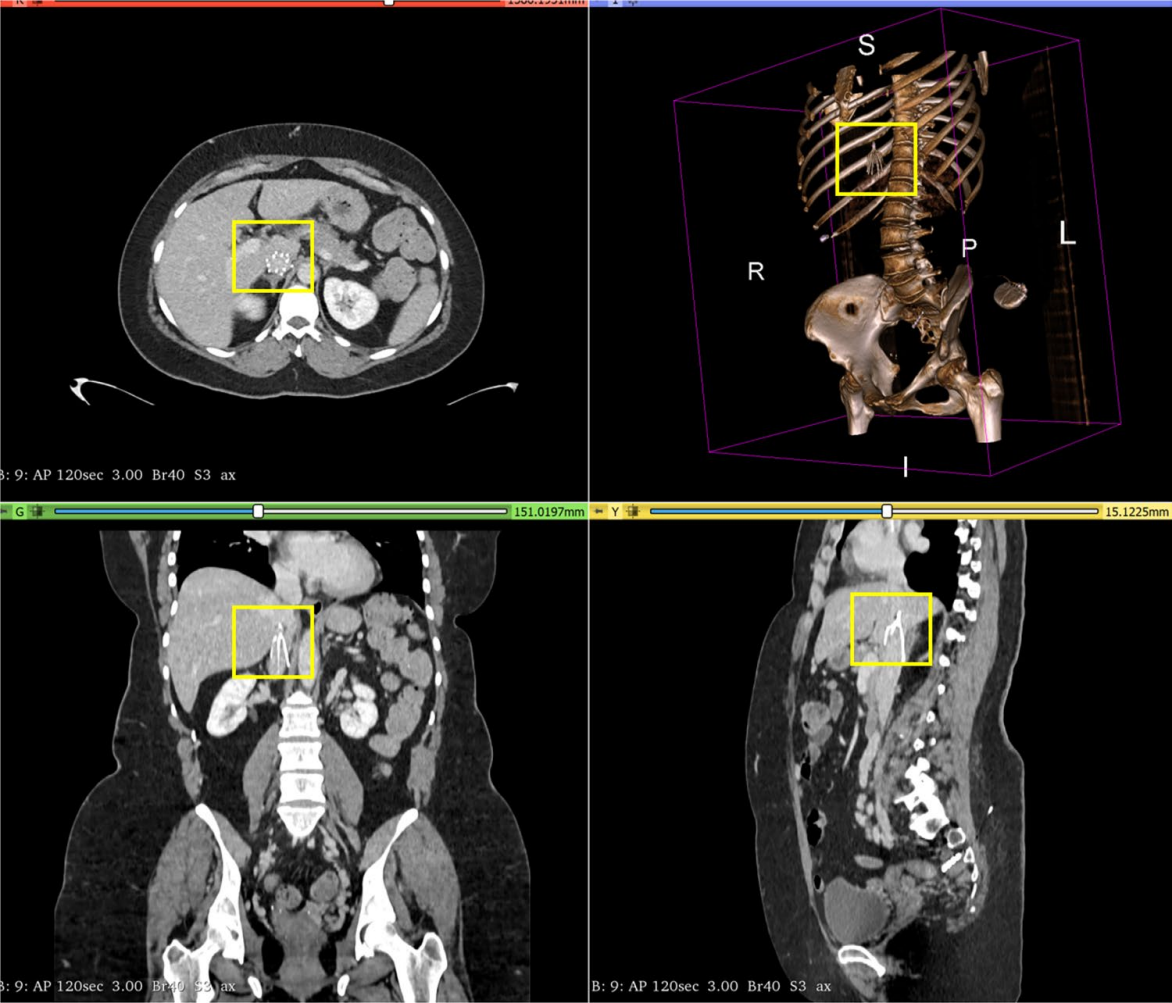
An inferior vena cava filter (indicated in yellow box)—axial, sagital, coronal views, and rendered volume. The figure is created using 3D slicer image computing platform [12]

The IVC, a large vein in the abdomen, is the primary conduit for blood from the lower body to return to the heart. This blood is then pumped into the lungs for oxygenation before returning to the heart to be pumped to the rest of the body. A blood clot in a vein may break off and travel through this route, where it lodges in the vasculature of the lungs, blocking blood flow and causing a pulmonary embolism (PE). The veins of the lower body, particularly the legs, have a greater propensity for blood clots, also known as deep venous thromboses (DVT). Conventional therapy to treat these clots is blood-thinners. However, some patients have other underlying medical conditions or require surgery, which makes the use of blood-thinners unsafe. In these circumstances, an IVC filter, which is a small metal device, needs to be placed in the IVC to stop any large clots from migrating towards the heart and lungs [13] (c.f., Fig. 1). Initially developed IVC filters were permanent, but modern filters are designed to be optionally retrievable once the acute risk of the clot has resolved [14, 15].

There are currently over a dozen FDA-approved IVC filters produced by different medical-device manufacturers. Additionally, several IVC filter types have been pulled from the market due to complications. Current guidelines recommend removing filters once they are no longer clinically warranted to reduce the risk of complications such as filter migration, device fracture, or erosion into adjacent tissue [13, 15]. Despite this, filter retrieval rates are as low as $8.5\%$ [16]. Patient loss to follow-up is an often-cited contributing factor for low retrieval rates. The detection of filters on imaging acquired for other indications, such as in the setting of trauma, can help improve follow-up rates by alerting medical providers, particularly in cases where patients are unsure of their medical history. Localization of filters plays an important role in assessing filter migration. Characterization of filter type is vital for detecting high-risk filter types that have been recalled and should be removed. Furthermore, characterization is needed to determine the feasibility of retrieval based on filter type. Currently, the detection and characterization of IVC filters are the roles of both diagnostic and interventional radiologists. Both traditional radiographs and CT scans can be used for this task, with CT scans providing more information. Whether or not a filter is retrievable can be determined by the morphological features of each filter type. In particular, the presence or absence of a hook at the superior pole of the IVC filter can be used to determine if it is of the retrievable type. This can often be a tedious task given the abundance of unique filter types.

**Fig. 2:**
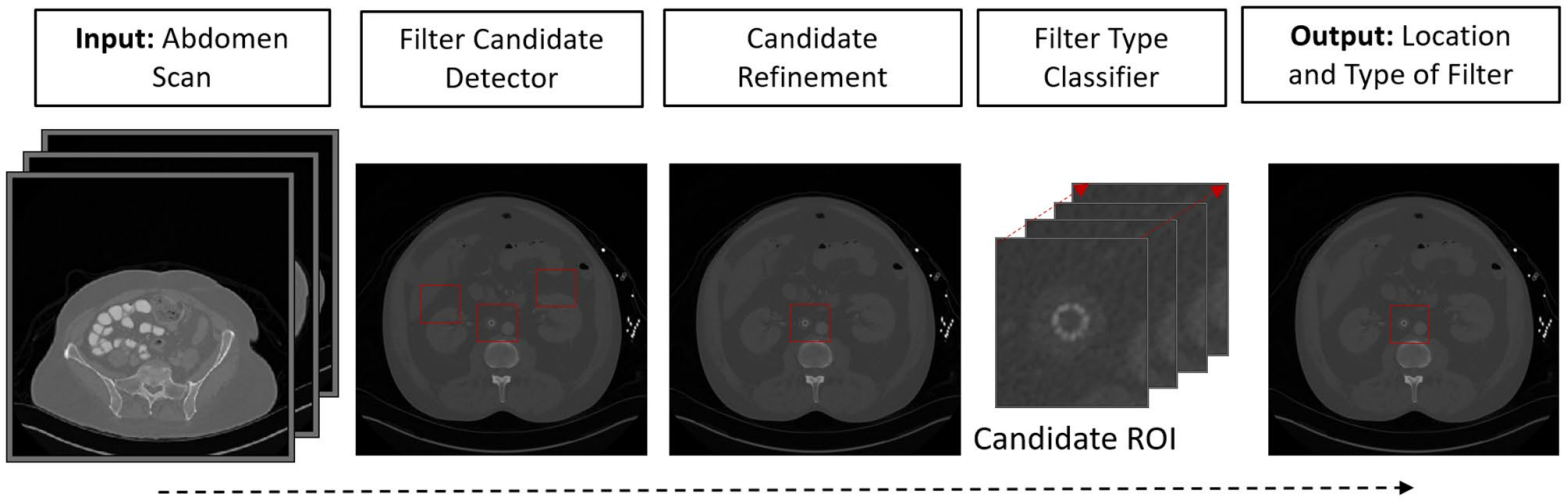
The diagram of IVC filter detection and classification system

This study proposes a three-stage data-driven computational framework to analyze abdominal CT scans for automated detection of IVC filters and determination of the type. The data-driven methodologies have been widely used in radiology [1]. In abdominal radiology, data-driven methodologies have been applied to organ segmentation, lesion detection, lung nodule detection, disease diagnosis, and disease grading [2, 17]. Based on the recent success of deep learning algorithms [18, 19], we hypothesize that a computational framework can learn the discriminative imaging characteristics of IVC filters, successfully locate them on CT scans, and reliably determine the filter type. The developed model might turn into a diagnostic decision support tool for interventional radiology and help manage clinical decisions. To our knowledge, there is only one study that investigated the automatic detection of IVC filter types [20]. The study focused on plain radiographic images, did not consider predicting filter presence or absence, did not provide filter localization, and worked solely on manually cropped regions. Unlike the previous work, our system is an end-to-end computational framework that processes abdominal computed tomography and provides filter presence prediction and localization stages. We have investigated the performance of two algorithms: one based on 2-dimensional data and transfer learning (2D+TL) [18] and an augmented version of the same algorithm which accounts for the 3-dimensional information leveraging recurrent convolutional neural networks (3D + RCNN) [21]. Figure 2 illustrates the overview of the proposed approach, which contains three main stages: detection of metallic object candidates (Section “Detecting Metallic Candidates”), IVC filter detection (Section “Detecting Filter Location”), and filter type classification (Section “Classifying Filter Type”).

## Materials and Methods

### Data

**Fig. 3:**
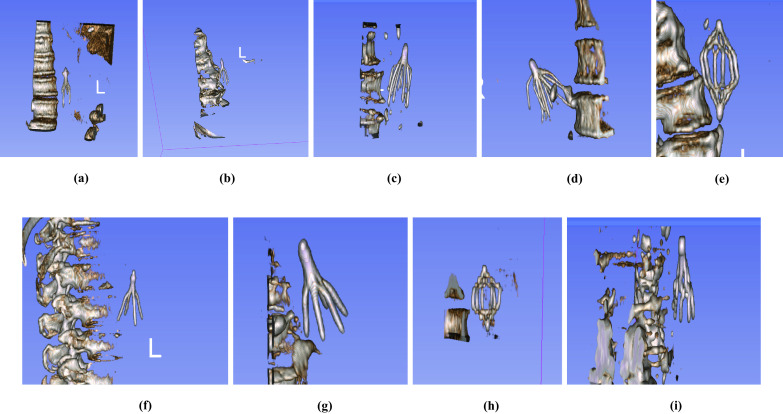
Rendered filter volumes. Filter types: **a** ALN, **b** Celect, **c** Denali, **d** G2, **e** OptEase, **f** Gunther-Tulip, **g** Option, **h** TrapEase (non-retrievable), **i** Greenfield (non-retrievable)

The study protocol was approved by the Institutional Review Board (IRB) with a waiver of consent, and all patient images were de-identified according to Health Insurance Portability and Accountability Act (HIPAA) rules. The initial inclusion criteria consisted of CT scans of adult patients who underwent IVC filter placement during a 10-year period from January 1st, 2009, to January 1st, 2019. Data were extracted from the picture and archiving communication system (PACS) of the Ohio State University Wexner Medical Center. A total of 2048 CT scans were obtained from 439 patients. Among them, 399 patients had retrievable filters, and 40 had non-retrievable filters (c.f., Table 1). Filter types in the development dataset included ALN (retrievable), Celect (retrievable), Denali (retrievable), G2 (retrievable), OptEase (retrievable), Gunther-Tulip (retrievable), Option (retrievable), TrapEase (non-retrievable), and Greenfield (non-retrievable) (c.f., Fig. 3).

### Reference Standards

We employed a supervised training strategy with a training set containing annotations that include the IVC filter type and their locations based on the 3D coordinates. The ground truth annotations of the filter types were determined based on the information available on the patient’s electronic medical record. The reference annotations of the filter locations were manually marked as rectangular bounding boxes on CT scans through an interface. We assumed the center of the bounding box being the center of the IVC filter object.

**Table 1:** Characteristics of the study subjects

Label	Info
# of Patients (PT)	439
# of Scans	2048
# of PT with retrievable filter	399
# of PT with non-retrievable filter	40
ALN (Retrievable)	8
Celect (retrievable)	38
Denali (retrievable)	315
G2 (retrievable)	1
Gunther-Tulip (retrievable)	3
Option (retrievable)	29
OptEase (retrievable)	5
Greenfield (non-retrievable)	37
TrapEase (non-retrievable)	3

### Detecting Metallic Candidates

The initial step of the framework is to locate the metallic candidates on abdominal CT scans. The inferior vena cava filter is a small metallic device. Given an abdomen CT scan, this stage of the algorithm returns a list of locations of the metallic regions based on the Hounsfield unit (*HU*), a measure of density on CT scans. The *HU* scale varies according to the density of the body part, such that below $-1000$ is considered air, $-500$ for lung, $+700$ to $+3000$ for bone, and over $+2000$ for metals. Instead of thresholding every slice in a CT with the Hounsfield scale, we first compute the CT scan’s maximum intensity projection (MIP) image. MIP is a computational process that projects the highest attenuation value along with one of the axis onto a two-dimensional (2*D*) image plane. We project the maximum intensity value of sequential abdomen scans on the axial plane to compute (*x*, *y*) coordinate of metallic object candidates on the coronal plane to calculate the corresponding *z* coordinate.

With $I^{MIP}$ representing the MIP image of a CT scan, and $I^{MIP}(x,y)$ representing the *HU* value at spatial location (*x*, *y*), we threshold the attenuation values of $\forall x \in I$ and $\forall y \in I$ in CT scans as follows:

1 $$\begin{aligned} I^{MIP}(x,y) = {\left\{ \begin{array}{ll} 0, &{} \text {if}\ I^{MIP}(x,y)<+2000 { HU} \\ I^{MIP}(x,y), &{} \text {if}\ I^{MIP}(x,y)>=+2000 { HU} \\ \end{array}\right. } \end{aligned}$$

The thresholding of the MIP image on the axial plane of a CT scan provides a list of (*x*, *y*) coordinates of metallic objects. The thresholding of the MIP image on the coronal plane provides the corresponding *z* coordinates of the metallic candidates. To assess the location of the candidates, we apply *connected component labeling*. Connected component labeling analyzes an image and groups the pixels based on the pixel neighborhood. Let $p \in I^{MIP}$ and $q \in I^{MIP}$ represent pixels. *S* is a connected component if there is a connected path from $\forall p \in S$ to $\forall q \in S$. We have used 8-*connectivity* to assign the labeling. Each connected component *S* is a cropped volume with a center coordinates (*x*, *y*, *z*). The later stage of the pipeline is classifying the candidates containing an IVC filter vs. not containing an IVC filter and locating the filter candidates with a confidence value. The candidates *S*, which contain a filter, are further processed for filter type determination.

### Data Processing

We applied pre-processing techniques before feeding the data to the data-driven models for filter vs. no filter classification. This stage aims to prepare the data to extract better representative discriminative features for each class and train the data-driven model more efficiently. We applied the following processing techniques:

*Data normalization*: This is the process of rescaling the intensity values to a range so that each training sample has a similar data distribution. We have applied *Z-score* normalization by subtracting the mean of the data from each instance and dividing the result by the standard deviation. The data normalization boosts the training performance and helps the classifier converge faster.

*Data augmentation*: Data-driven methods require large amounts of data to train a model. If the data is limited, the data-driven model may suffer from overfitting, which results in poor generalizability. We have applied data augmentation that increases the size of the data and the variability in appearance [19]. We randomly shifted CT scans along *x*, *y*, and *z* axes with [-3, 3] pixels and rotated randomly between 2 and 5°.

### Detecting Filter Location

**Fig. 4:**
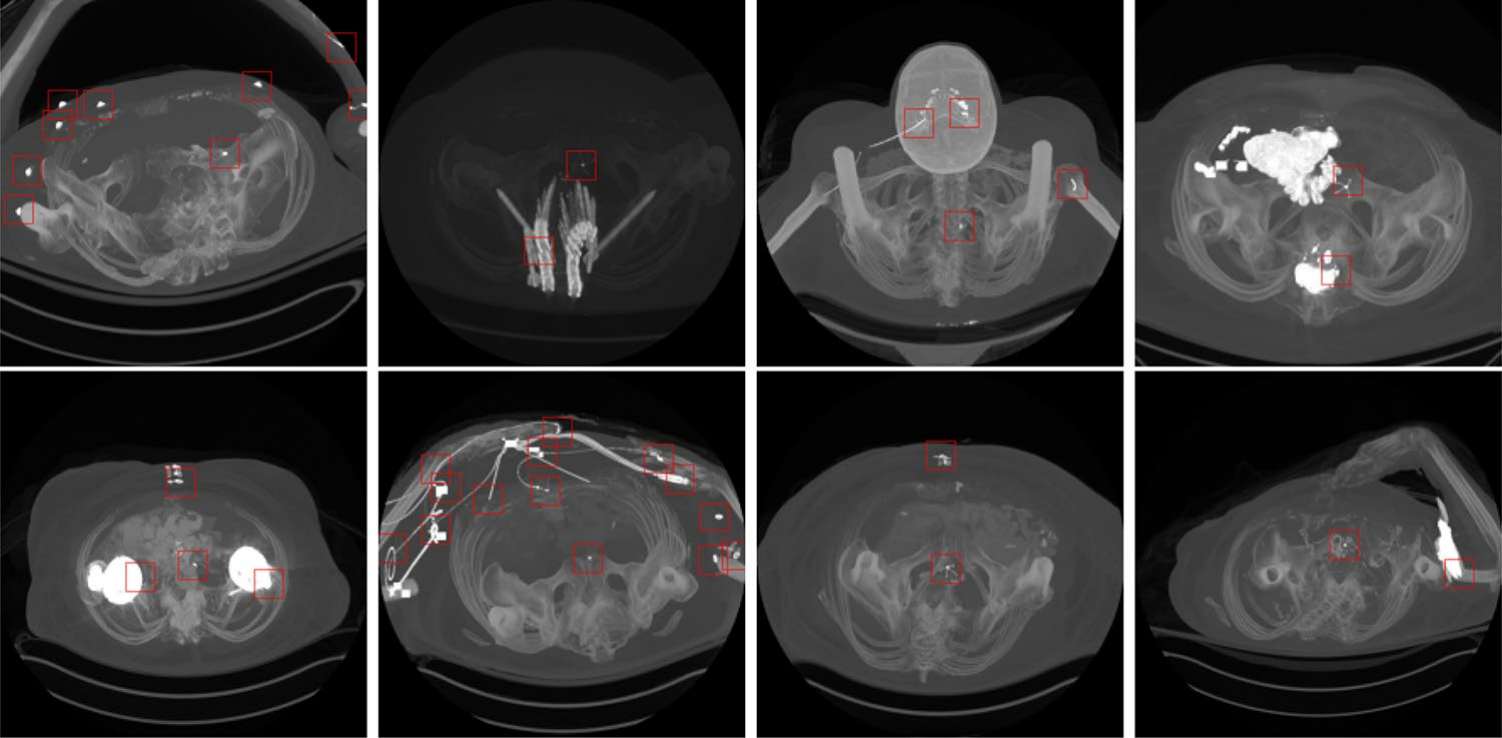
Example metallic objects in the abdominal CT (indicated in red bounding box), which have higher attenuation value than the predetermined threshold attenuation value for metallic objects. Several metallic objects can be present in the abdominal CT scan, such as surgical clips, intravascular stents, spinal fusion hardware, retained shrapnel, or swallowed foreign-bodies

The previous step of the framework provides metallic object candidates *S*, which have higher attenuation values than the predetermined threshold for the metallic objects. Several locations may contain metallic objects in the abdominal CT scan, such as surgical clips, intravascular stents, spinal fusion hardware, retained shrapnel, or swallowed foreign bodies (c.f., Fig. 4). However, the appearance of these candidates is different from the appearance of an IVC filter. We have utilized data-driven models which process candidate regions, eliminate non-filter metallic objects based on appearance features, and predict the IVC filter location. We have developed two data-driven models for this task. First, a two-dimensional (2D) convolutional neural network (CNN) was trained to refine the candidates. Later, we have trained a recurrent CNN to use the spatial knowledge between the sequential slices. We formulate the filter refining stage as a binary classification problem. We aim to find a function $y=f(S)$ where the input is the metallic volume *S* extracted from CT scans at Section “Detecting Metallic Candidates”, and the output is a binary label $y \in {0,1}$ indicating the region of interest contains an IVC filter or not.

Given a set of metallic candidate locations with corresponding true label, we build a training dataset $R = \{S_i, y_i\}_{i=1}^N$ where $S_i$ is a candidate metallic volume at coordinate ${(x^c_i, y^c_i, z^c_i)}$, $y_i$ is the true label and indicating the region of interest $S_i$ contains an IVC filter or not, and *N* is the number of training samples. The networks are trained with Adam optimizer [22] by minimizing the binary cross entropy

2 $$\begin{aligned} H_p(q) = -\frac{1}{N}\sum _{i=1}^{N}y_i\log (p(y_i))+(1-y_i)\log (1-p(y_i)) \end{aligned}$$

where $p(y_i)$ is the predicted probability that the $S_i$ contains filter.

We have used data-driven models to process the metallic volumes *S* and predict those with IVC filters. The data-driven models are based on CNN, which hierarchically extracts representative features by processing image sequences with kernel filters [18, 23]. The strengths of CNNs are their ability to learn the internal representation of images and preserve the local connectivity that allows the architecture to learn the spatial pattern. To our knowledge, CNNs have not been used to analyze CT scans for IVC filter detection and filter type classification. We expect the system to learn the filter’s location and appearance during the model training stage.

**Fig. 5:**
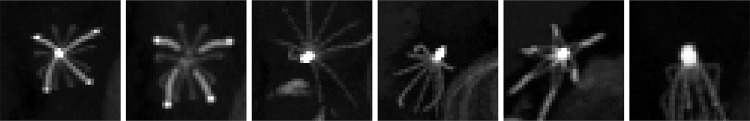
MIP images of region of interests $S_i$ which contain a filter

**Fig. 6:**
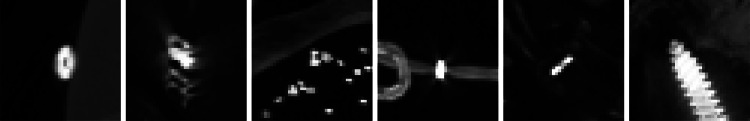
MIP images of region of interests $S_i$ which have higher attenuation values but do not contain a filter

We first developed a *2D-CNN* to classify the candidate volumes *S* for IVC filter detection. We used the VGG-16 [24] as a backbone architecture, removed its fully connected layers, and inserted a fully connected layer, a dropout layer with a parameter of 0.5, and an output layer with two nodes representing the probability of *S* containing an IVC filter. Although we have been working with a relatively large dataset, the number of scans is still limited for training a model from scratch (c.f., Table 1). Therefore, we utilized the fine-tuning training strategy in which the machine learning model initialized with weights pre-trained on another dataset. For the study, the weights are pre-trained on ImageNet [23]. To feed the 2D CNN architecture with volume *S*, we computed the maximum intensity projection (MIP) image of the region of interest *S*. Figures 5 and 6 show MIP images of example region of interest $S_i$ which contain a filter and MIP images of example region of interests $S_i$ which have higher attenuation values but do not contain a filter, respectively.

Analyzing the volumetric data with a 2D-CNN using MIP images may lead to loss of volumetric information, which is the temporal relationship between the slices. To incorporate the temporal knowledge in the training process, we have developed a *recurrent CNN* architecture which is the combination of recurrent NN and CNN. The recurrent neural networks (RNN) have the ability to process and learn features from sequential data. The combination of CNN and RNN builds a hybrid model that captures both temporal and spatial features in the data. The abdominal CT scans can be processed as sequential data. The change throughout the scan is the temporal behavior. Therefore, in our setting, the temporal dimension is substituted with the third dimension (*z*-axis). We built a recurrent CNN model and processed the volume *S* for filter localization and filter type classification. The backbone CNN architecture of the recurrent CNN is the VGG-16 [24]. The model also utilized the fine-tuning strategy to initialize the weights of VGG-16. We extract the imaging features of each scan through the CNN that learns the spatial knowledge. We then process the extracted features through an RNN, and that learns the temporal behavior of *S*. The RNN consists of two layers of 16 and 8 gated recurrent units (GRUs) with a dropout layer between GRU layers with a parameter of 0.5 and an output layer with two nodes representing the probability of *S* containing an IVC filter.

### Detecting Filter Location—Post-Processing

The filter detection component of the system predicts the location of the IVC filter and locates it with a bounding box (BB) with an associated confidence value. The softmax probability of the models is considered model confidence for its decision for the assigned label. One of the issues in the object detection systems is overlapping bounding boxes that refer to the same object. To remove the repetitive bounding boxes, we have applied *Non-Maximum Suppression* which selects the best bounding box for objects by taking into account the confidence value predicted by the model and overlapping score of bounding boxes.

### Classifying Filter Type

This stage of the system processes the predicted filter locations and outputs the probability of filter types as retrievable vs. non-retrievable. Accurate IVC filter type identification is a challenging object recognition problem due the minor variations in appearance between the filter types (c.f., Fig. 3). Additionally, the number of instances for some subtypes of the filters is not enough to train a robust classifier (c.f., Table 1). Therefore, instead of applying a multi-class classification, we train our model to separate the filters into two classes as retrievable vs. non-retrievable cases. We utilize the same architectures developed in filter localization stage, 2D CNN, and recurrent CNN, which are defined in Section “Detecting Filter Location—Post-Processing”. We trained the models with retrievable and non-retrievable filters. Therefore, the models learn the morphological differences of filter types and determine the subtype.

## Experiments

**Fig. 7:**
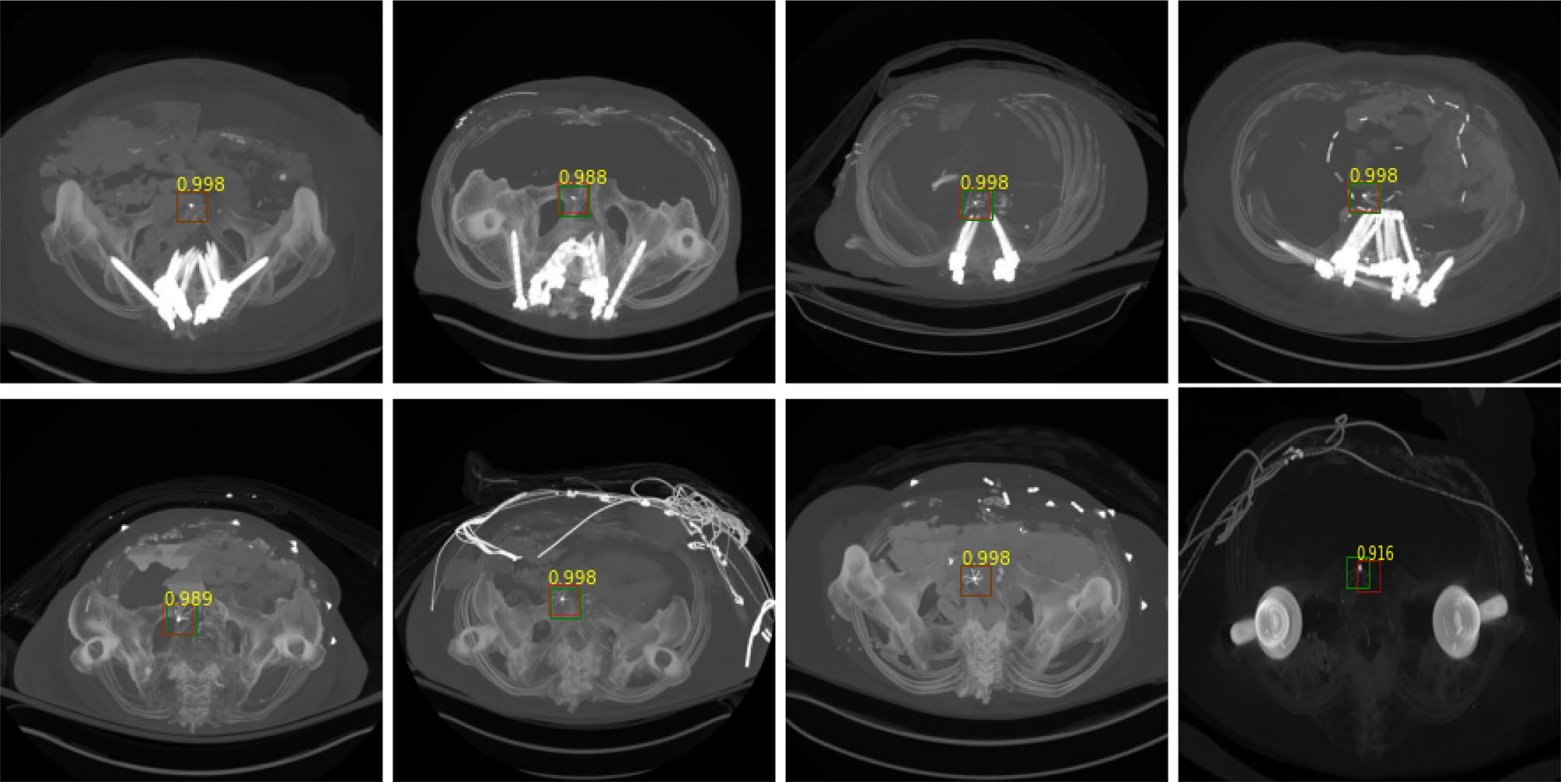
Instances that IVC filters are detected accurately (TP). Green bounding box (BB) is the ground truth, red BB is the prediction, and associated yellow text is the confidence of the prediction. The performance of detection stage is measured based on bounding boxes overlap (c.f., Table3)

This section contains the quantitative analysis of the model performance during training and validation. The models are developed in Python using Tensorflow Keras API and trained on the Nvidia Quadro GV100 system with 32GB graphics cards with CUDA/CuDNN v9 dependencies for GPU acceleration. The data were randomly shuffled and partitioned into training, validation, and test sets (c.f., Table 2). The split is conducted at the patient level, so the same patient scans are located in the same set. We ensured that there is no patient overlap between the splits (Fig. 7).

### Filter Detection

**Fig. 8:**
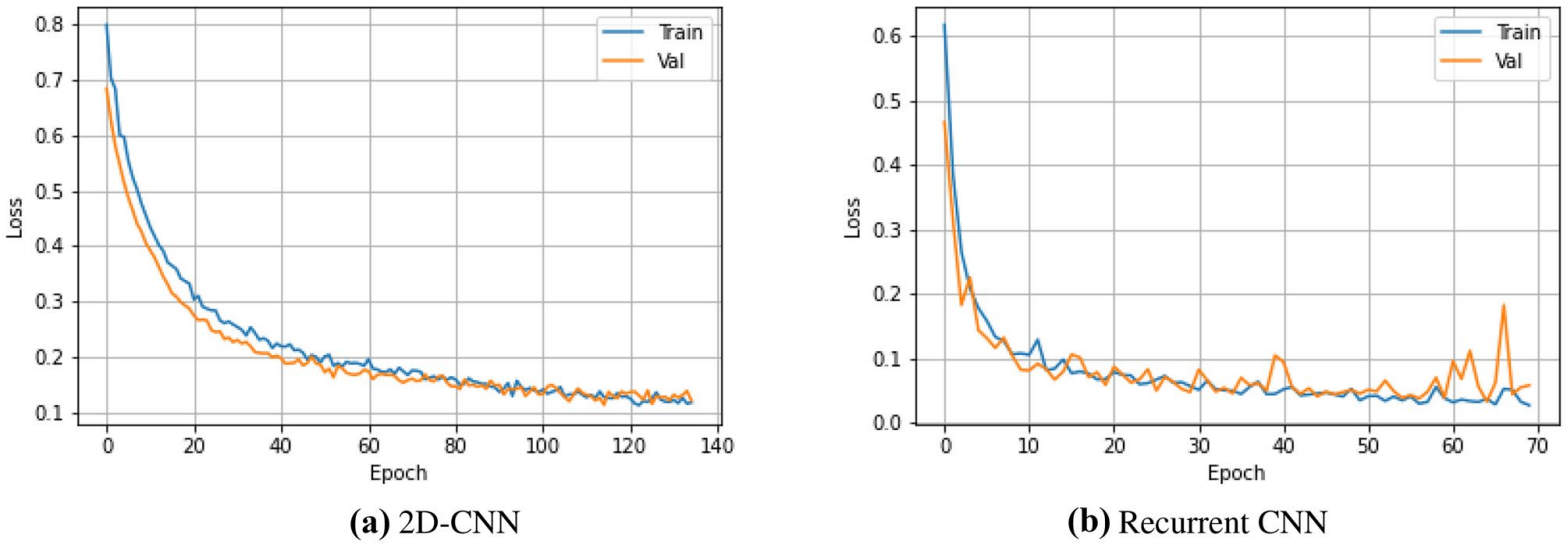
Model development stage: filter candidate localization—training and validation loss with **a** 2D-CNN **b** recurrent CNN

The filter detection component of the system predicts the IVC filter presence or absence and locates the filter with a bounding box (BB) with an associated confidence value. For this stage, both 2D-CNN and recurrent CNN models were used. The parameters of the classification networks and training protocol are listed in Table 2. The optimal hyper-parameters were experimentally determined. The binary-cross-entropy loss and Adam optimizer [22] were used with default parameters of $\beta _1$ = 0.9 and $\beta _2$ = 0.999. The learning rate was fixed at $1 \times 10^{-4}$ during the training. The models were trained until validation loss failed to improve (Fig. 8).

**Table 2:** Filter candidate localization stage. Parameters used on both 2D and 3D of the classification neural networks and training protocol

Parameter	Value
# of patients in training set	109
# of scans in training set	1076
# of patients in test set	329
# of scans in test set	972
*HU* threshold for metals	2000
Threshold for confidence value	0.7
Threshold for BB overlap	0.5
Loss function	Binary cross entropy
Optimizer	Adam with $\beta _1 = 0.9$ and $\beta _2 = 0.999$
Learning rate	Fixed $1 \times 10^{-4}$
Number of batch	16
Backbone architecture	VGG-16
Training strategy	Fine-tuning

In order to determine how well the system predicts the location of the IVC filters, the predicted locations are compared with the reference locations by measuring the bounding box (BB) overlaps. We have used the intersection over union threshold (IoU) metric to decide how well the predicted BB overlaps with the reference filter location. A detection is considered successful if the intersection between the BB is $>=0.5$, which is labeled as true positive (TP). If BB does not contain an IVC filter, it is considered misdetection, false positive (FP). If the model fails to locate the IVC filter, it is false negative (FN). True negative (TN) is every part of the image without filter, where the model did not predict the filter. The quantitative results on test set are listed in Table 3. We found that the system achieved high sensitivity on detecting the filter locations. Example qualitative results are shown in Fig. 7.

**Fig. 9:**
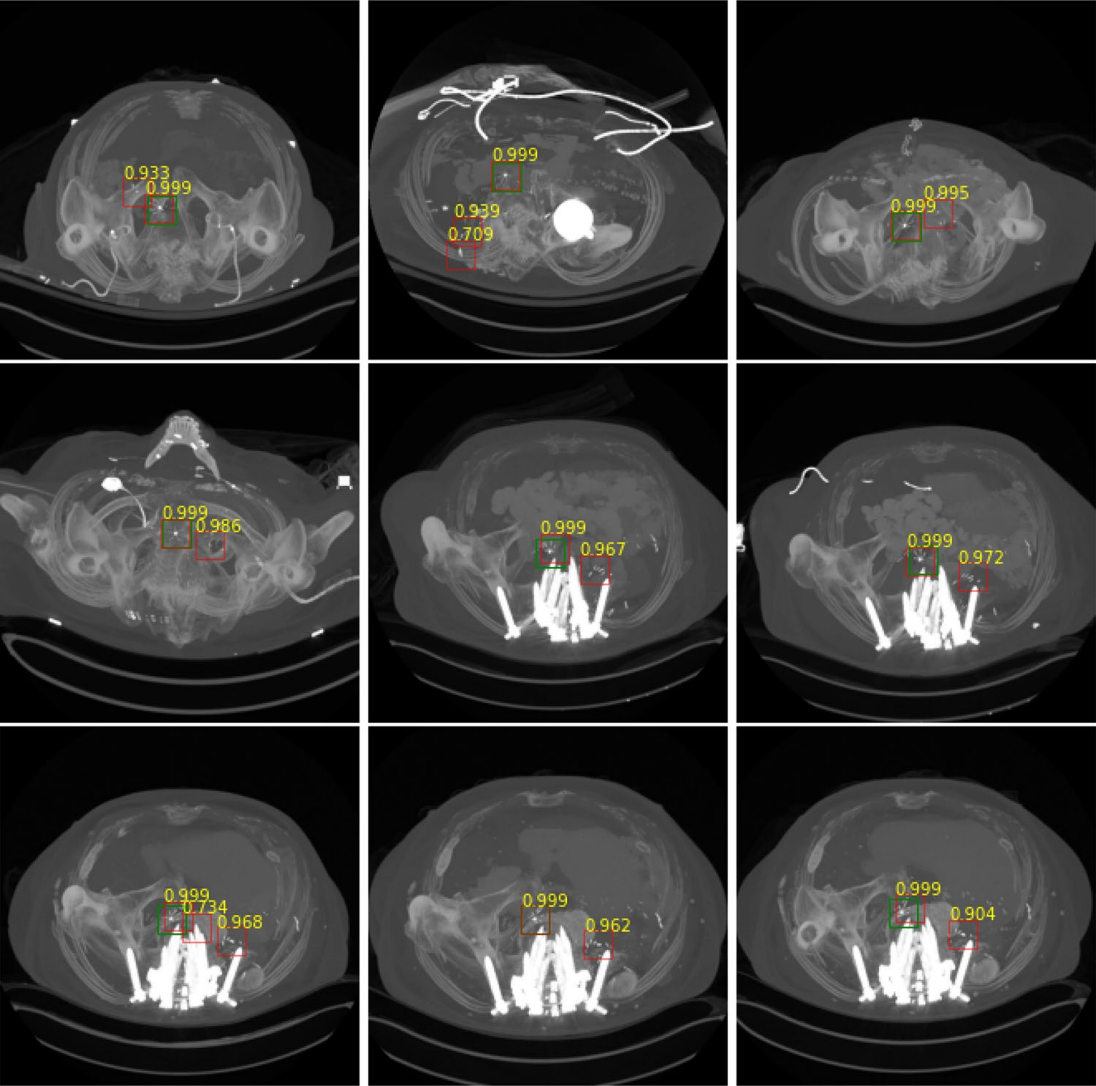
Instances with FP cases. Green bounding box (BB) is the ground truth, red BB is the prediction, and yellow text is the confidence of the prediction. Note that the model confidence for the FP predictions are lower than the TP predictions

The filter detection module processes *S* regions and predicts if it contains an IVC filter. The softmax probability of the module is considered model confidence for its decision for the assigned label. Any prediction with a confidence higher than 0.7 are considered filter (c.f., Table 2). Figure 9 shows cases that the system incorrectly labeled additional regions without a filter (False Positives). However, note that the system predicts the filter area correctly with a higher confidence value.

**Table 3:** Evaluation: filter candidate localization with 2D-CNN and recurrent CNN

Method	Metric	Value
2D CNN	Recall	0.911
	Precision	0.804
	F1	0.854
	Avg. confidence for TP	0.993
Recurrent CNN	Recall	0.923
	Precision	0.853
	F1	0.886
	Avg. confidence for TP	0.996

In order to measure the filter detection component performance for filter presence/absence prediction, we run the model on a control dataset which contains patient scans without any IVC filter. We expect the system not to detect any location if the abdomen CT does not have an IVC filter. The control dataset contains 27 patient data with 91 abdomen scans. The system was successful on 90 scans and did not predict any location. The system located a false positive filter area on one scan which does not contain an IVC filter.

### Filter Type Prediction

**Fig. 10:**
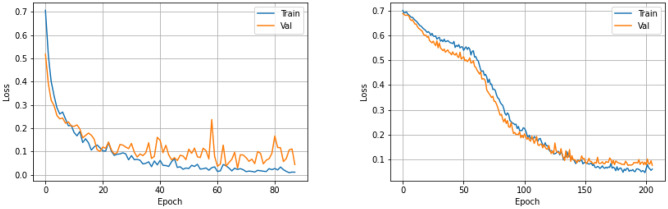
Model development stage: filter type classification—training and validation loss with 2D-CNN (left graph), recurrent CNN (right graph)

**Fig. 11:**
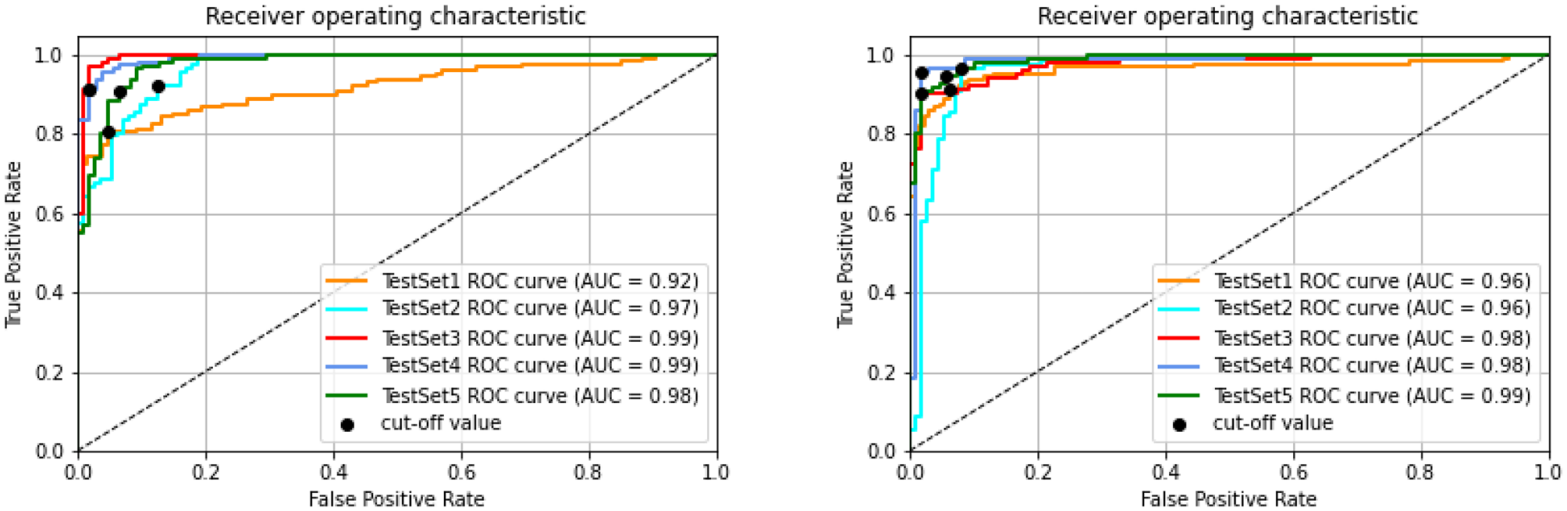
Filter type classification—evaluation on test data for retrievable vs. non-retrievable classification. 2D-CNN (left graph), recurrent CNN (right graph)

The filter type detection component of the system predicts whether the filter type is retrievable or non-retrievable. For this stage, we process the *S* volume with 2D-CNN and recurrent CNN models, with $VGG-16$ backbone architecture and fine-tuning strategy. The parameters of the classification networks and training protocol are listed in Table 4. The optimal hyperparameters were experimentally determined. The binary-cross-entropy loss and Adam optimizer [22] were used with default parameters of $\beta _1 = 0.9$ and $\beta _2 = 0.999$. The learning rate was fixed at $1 \times 10^{-5}$ during the training. We approach the filter type labeling as a binary classification. The evaluation metrics for binary classification (for our problem, it is assessing the filter type) are sensitivity, specificity, accuracy, positive and negative predictive probability, and area under the curve scores. The receiver operating characteristic (ROC) curve provides the model performance at various thresholds. We have computed the cut-off value (optimal decision threshold) on the validation set, which has a high true positive rate and a low false-positive rate (Figs. 10 and 11). The model performance for the optimal decision threshold is listed in Table 5.

**Table 4:** Parameters of the filter type detection networks and training protocol

Parameter	Filter type
# of samples for retrievable type	563
# of samples for non-retrievable type	524
*HU* threshold for metal and tissue around	0
Loss function	Binary cross entropy
Optimizer	Adam with $\beta _1 = 0.9$ and $\beta _2 = 0.999$
Learning rate	Fixed 0.00001
Number of batch	16
Backbone architecture	VGG-16
Training strategy	Fine-tuning

**Table 5:** Filter type classification performance on test data for the optimal decision threshold (cut-off value)

Metric	2D-CNN	Recurrent CNN
Accuracy	0.913	0.933
Precision	0.888	0.928
Sensitivity	0.945	0.940
Specificity	0.882	0.927
NPV	0.945	0.941
FPR	0.117	0.073
F1 score	0.915	0.933
AUC	0.970	0.975

### Generalization Performance of the System

We have curated an additional set of 200 examinations, 100 of which contained IVC filters and 100 of which did not. The examinations containing IVC filters were manually labeled as either retrievable or non-retrievable by reviewing the corresponding medical records. Initially, the examinations were processed through the filter detection component of our system to identify the filter candidates. Examinations that yielded no candidates according to the model were considered having no filter. Conversely, examinations producing one or more candidates were classified as having a filter according to the model. As the model can generate multiple candidates for each examination and is designed to function independently without human intervention, we opted to automatically select the candidate with the highest confidence score assigned by the model. These candidates were then evaluated in the subsequent filter type identification stage. The results are reported in Table 6.

**Table 6:** Generalization performance of the system on unseen data. *NPV*, negative predictive value; *PPV*, positive predictive value

Task	Accuracy	Sensitivity	Specificity	NPV	PPV	F1 score
Filter detection	0.855	0.760	0.950	0.938	0.798	0.84
Filter type classification	0.750	0.852	0.579	0.807	0.579	0.829

## Conclusions

Data-driven approaches have demonstrated advancements in recent years in computational framework development in healthcare. The developed frameworks learn the underlying pattern from large-size imaging data and make predictions for the objective healthcare problem. In this study, we proposed a three-stage data-driven approach that analyzes abdominal CT studies to predict IVC filter presence, automated detection of the filter location, and determination of the filter type. The proposed framework utilized 2-dimensional- and recurrent convolutional neural networks with VGG-16 as backbone architecture. The data-driven models successfully learned the morphology of the IVC filters, predicted the presence of a filter on abdominal CT, correctly located it on the scan, and determined the filter type. The system achieved high sensitivity on detecting the filter locations with high confidence. The 2D-CNN model achieved a sensitivity (true positive rate) score of 0.911 and precision score of 0.804 for filter detection. The recurrent CNN model achieved a sensitivity score of 0.923 and a precision score of 0.853 for filter detection. The confidence value reflects the certainty in the prediction. The system confidence for the predictions is 0.993 for 2D-CNN and 0.996 for recurrent CNN. The filter detection component also achieved a high precision score (0.989 with recurrent CNN) on the control dataset, containing patient scans without any IVC filter. Accurate IVC filter type identification is a challenging object recognition problem due to minor appearance variations between the filter types. We designed the filter type determination component as a binary classifier that predicts the filter type as retrievable vs. non-retrievable. The system achieved 0.945 sensitivity, 0.882 specificity, and 0.970 AUC score with 2D-CNN for filter type classification. The system achieved 0.940 sensitivity, 0.927 specificity, and 0.975 AUC score with recurrent CNN model.

The primary purpose of AI-based research in medical imaging is to create tools that improve patient outcomes [25]. This study is the initial phase of a computational framework which has a potential to turn into a diagnostic decision tool that may support radiologists in locating the filter, detecting the filter type, and helping them with filter management. Our proposed framework is designed to be utilized in conjunction with radiologist, ensuring that they are always involved in the diagnostic process. With the radiologist in the loop mitigates concerns associated with potential errors.

In this study, we have developed the initial phase of our model for filter location detection and filter type prediction on CT scans. The study involved adult patients who underwent IVC filter placement at the Ohio State University Wexner Medical Center over a 10-year period, from January 1st, 2009, to January 1st, 2019. However, we recognize the potential future data shifts, which may arise from factors such as using new devices and changes in acquisition protocols. Data shift can alter the distribution of the data on which the model is trained consequently lead to a decline in model performance. Therefore, it is crucial to periodically re-evaluate the model and adapt it accordingly when faced with data shifts. Retraining the model using newly curated data obtained from the new device can address this issue. By combining this new data with the existing dataset, the model’s weights can be updated to capture the characteristics of the new device’s data. The other approach would be employing online learning, where the model’s weights are incrementally fine-tuned with the new data while utilizing old weights as initial stage for the optimization process.

We also would like to highlight the persistent value of our detection model as IVC filter technology evolves. From 1980 to 2014, there have only been unique 23 filter types passing FDA clearance [26]. The rate of new designs is slow, and all the 510k FDA approvals for IVC filters in the last 20 years involved modifications to previously established filter design, such as the Greenfield, Celect, Tulip, Bird’s Nest, Optease, and Option Filter [27]. Novel filter designs adhere to stringent standards, encompassing the effective management of small puncture sites, maintenance of caval patency, avoidance of cava perforation, biocompatibility, non-thrombogenicity, compatibility with magnetic resonance imaging, and resistance to fracturing or migration. Furthermore, there is additional market demand for new IVC filters to be optional with the possibility for intravascular removal. Even with new designs, the rate of adoption will be slow, as physicians have preferential use of previously established filter types, as these have the most evidence-based support for effectiveness and predictable complication rates. Our results show that we can detect these most common filter types with robust accuracy. Accounting model update strategies and considering the slow-paced evaluation of IVC filter technology, our IVC filter detection algorithm will maintain relevance and utility for many years and can adapt itself over time to ensure performance remains accurate and reliable.
